# The novel coronavirus (COVID-19) pneumonia with negative detection of viral ribonucleic acid from nasopharyngeal swabs: a case report

**DOI:** 10.1186/s12879-020-05045-z

**Published:** 2020-04-30

**Authors:** Peiyan Zhang, Zhao Cai, Weibo Wu, Ling Peng, Yinfeng Li, Chuming Chen, Li Chen, Jianming Li, Mengli Cao, Shiyan Feng, Xiao Jiang, Jing Yuan, Yingxia Liu, Liang Yang, Fuxiang Wang

**Affiliations:** 1 Shenzhen Third People’s Hospital, Second Hospital Affiliated to Southern University of science and Technology, Shenzhen, Guangdong Province China; 2 School of Medicine, Southern University of Science and Technology, Shenzhen, 518055 Guangdong Province People’s Republic of China

**Keywords:** Coronavirus, COVID-19, Pneumonia, Tracheoscopy, Bronchoalveolar-lavage fluid

## Abstract

**Background:**

The novel coronavirus disease 2019 (COVID-19) outbreak started in Wuhan, Hubei, China since Dec 2019 and cases of infection have been continuously reported in various countries. It is now clear that the SARS-COV-2 coronavirus is transmissible from human to human. Nucleic acid detection is considered as the gold standard for the diagnosis of COVID-19. In this case report, we describe our experience in detection of SARS-COV-2 from a confirmed patient using nucleic acid test of bronchoalveolar-lavage fluid (BALF) samples but not nasopharyngeal swabs.

**Case presentation:**

We present a case of severely ill SARS-COV-2 infected 46-year-old man with fever, coughing and chest tightness. We performed viral detection using his BALF samples and imaging method (CT) for confirmation. The patient received combination of interferonalfa-1b and ribavirin, lopinavir and ritonavir for antiviral treatment at different stages. Other medication was also given to him in combination for anti-inflammation, intestinal microbial regulation, phlegm elimination, liver protection and pulmonary fibrosis prevention purposes. We provided oxygen supply to him using BIPAP ventilator and high-flow humidification oxygen therapy instrument to facilitate respiration. The patient was cured and discharged.

**Conclusion:**

This case report described an effective supportive medication scheme to treat SARS-COV-2 infected patient and emphasized the necessity of detection of the viral genome using BALF samples and its significance in the diagnosis and prognosis of the disease.

## Background

The outbreak of the novel coronavirus disease COVID-19 caused by severe acute respiratory syndrome coronavirus 2 (SARS-COV-2) started in Wuhan, Hubei, China from December 2019, and has been spread out to the world [1, 2]. As of April 10, 2020, there are 83,323 confirmed cases in China among which 3346 died, while more than 1,527,482 confirmed cases in the world among which 92,532 died [3]. It is speculated that the natural hosts of SARS-COV-2 are possibly wild animals such as bats and pangolins [4, 5]. However, this is still under discussion. The virus could spread from human to human through aerosol, facal-oral route and eye secretions [6–9]. Further understanding on its epidemiology, transmission mechanism and spectrum of symptoms is under continuous investigation. This case report describes the symptoms, diagnosis and treatments of one unconventional COVID-19 pneumonia case in our hospital during early period of the outbreak when having very little understanding on the virus and the infection.

## Case presentation

On 23 January 2020, a 46-year-old man was transferred to our hospital with 11-days history of fever of 38 °C and coughing. The patient permanently resides in Yili Development Zone, Xinjiang, China. He started low fever with dry cough, muscle ache and fatigue without known causes in Yili on 12 January. The patient disclosed that he had been in close contact with a person from Wuhan in Yili on 10 January and had a travel history with flights to Shanghai on 16 January 2020, from Shanghai to Ningbo on 17 January 2020, and from Ningbo to Shenzhen on January 19, 2020, without traveling or living history to Wuhan. On 18 January, there was onset of symptoms including chest tightness without chest pain and hemoptysis. Unknown medication was taken by the patient without symptomatic improvement. He was considered as a pneumonia patient and admitted to the University of Hong Kong-Shenzhen Hospital on 19 January. Results of blood gas analysis showed a pH of 7.445, carbon dioxide partial pressure of 4.72 KPa and oxygen partial pressure of 7.82 KPa. He was tested negative for influenza A/B virus and respiratory syncytial virus (RSV), *Mycoplasma pneumoniae*, *Cryptococcus haemolyticus* antigen, *Aspergillus* antigen, Epstein-Barr virus capsid antigen IgM, Epstein-Barr virus DNA, Cytomegalovirus DNA and antigen IgM. He was isolated in a single ward and received oxygen support and levofloxacin treatment. On 22 January, the patient was still having fever of 38.5 °C with chest tightness and shortness of breath. BALF was tested negative for *Aspergillus*, *Legionella*, *Pneumocystis carinii*, acid-fast *Bacilli*, *Mycobacterium tuberculosis*. He was diagnosed with severe bilateral community-acquired pneumonia (not excluding the possibility of COVID-19 pneumonia) and hypoxemia. Levofloxacin treatment was thus stopped and changed to combined anti-infection treatment of amoxicillin and clavulanate potassium and doxycycline. He was transferred to the Third People’s hospital of Shenzhen for further treatment on 23 January. The patient has no history of other diseases, surgical trauma, food and drug allergy. There was no headache, dizziness, vomiting, abdominal pain, diarrhea, frequent urination, urgent urination, or urination pain claimed by the patient.

The results of physical examination on 23 January showed a body temperature of 36.1 °C, pulse of 94 times/min, respiratory rate of 26 breaths/min and blood pressure of 127/87 mmHg. Clinical laboratory test results revealed negative results for mycoplasma, chlamydia, cytomegalovirus-IgM, influenza A/B virus and RSV. Throughout the whole period of hospitalization, the patient was isolated in a single ward and given 60 μg of interferonalf-a1b (Beijing Tri-Prime Gene Pharmaceutical Co., Ltd., China; Shenzhen Kexing Biopharm, China) inhalation for antiviral purpose, 0.4 g of Bio-Three tablets (Huizhou Jiuhui Pharmaceutical Co., Ltd., China; Toa Pharmaceutical Co.,Ltd.Tatebayashi Plant, Japan) and 420 mg Bifid-triple viable capsule (Inner Mongolia Shuangqi Pharmaceutical Co., Ltd., China) three times a day for regulation of intestinal microbiome, and 30 mg mucosolvan (Boehringer Ingelheim Espana,S,A.) intravenous injection twice per day for phlegm elimination. A 3-day course of 0.5 g ribavirin (Jiangsu Lianshui Pharmaceutical Co., Ltd., China) intravenous injection starting from 23 January was also given to the patient in combination with Interferonalfa-1b twice a day for 3 days for antiviral treatment of RNA virus. (Fig. 2).

On 24 January, the patient was reported to have shortness of breath with respiratory rate of 38 times/min and heart rate of 90 times/min. Blood gas analysis revealed a pH value of 7.428, carbon dioxide partial pressure of 43.0 mmHg, oxygen partial pressure of 64.4 mmHg, actual bicarbonate level of 28.4 mmol/L, oxygen saturation of 92% and fractional concentration of inspired oxygen (FiO_2_) of 41.0%. (Table 1) Supplemental oxygen was applied to the patient with non-invasive BIPAP ventilator using IPAP 14cmH_2_O and EPAP 7cmH_2_O with oxygen concentration of 45%. His respiratory rate was 16 times/min after receiving oxygen supplement. Shortness of breath was gradually relieved and patient’s oxygen saturation values of peripheral blood reached 99 to 100%. Routine blood test revealed a white blood cell count of 5.61 × 10^9^/L with 80.40% neutrophil and 14.80% lymphocyte, hemoglobin concentration of 158 g/L, platelet count of 207 × 10^9^/L (Table 1) and erythrocyte sedimentation rate (ESR) of 71 mm/h. Biochemical test results showed an elevated D-Dimer (diffuse intravascular coagulation, DIC) level (Table 1) which may induce thrombus. 0.4 ml of nadroparin calcium (ASPEN Notre Dame de Bondeville, France) subcutaneous injection was given to the patient once per day for anti-coagulation until 31 January. D-DIC level decreased to normal range at 26 January. The patient also received 40 mg of esomeprazole sodium (AstraZeneca Pharmaceutical Co.,Ltd.) intravenous injection once per day for gastro-esophageal reflux suppression until 30 January and 30 mg of methylprednisolone (Pfizer Manufacturing Belgium NV) intravenous injection once every 12 h until 28 January for anti-inflammation treatment. The increase in white blood cells (Table 1) during this period may be due to the effect of methylprednisolone. However, COVID-19 ribonucleic acid test was negative using nasopharyngeal swabs done by Shenzhen Center for Disease Control (Shenzhen CDC). Treatment scheme and viral detection time points are illustrated in Fig. 2.

**Table 1:** Laboratory test results. Elevated level is indicated by bold and decreased level is indicated by underline

Tests		Normal range	24-Jan	25-Jan	26-Jan	27-Jan	28-Jan	29-Jan	30-Jan	31-Jan	1-Feb	2-Feb	7-Feb	12-Feb
Routine Blood Tests	white blood cell count (10^9^/L)	3.5–9.5	5.61	4.59	–	**9.89**	9.48	7.41	–	7.02	–	–	5.12	3.63
	Neutrophil ratio (%)	40–75	**80.4**	73.1	–	**87.4**	**88.3**	**80.1**	–	**78.2**	–	–	**75.7**	54.2
	Lymphocyte ratio (%)	20–50	14.8	18.7	–	7.9	7.1	11.3	–	13.4	–	–	13.5	32.8
	Eosinophil ratio (%)	0.4–8	0.5	0.2	–	0	0	0	–	0.3	–	–	1	2.5
	T cell ratio (%)	65–79		–	–	–	–	49.1	–	–	–	–	–	66
	T cell absolute count (/μl)			–	–	–	–	337	–	–	–	–	–	920
	Th-cell absoluet count(/μl)			–	–	–	–	223	–	–	–	–	–	534
	Ts-cell absolute count(/μl)			–	–	–	–	110	–	–	–	–	–	379
	Th/Ts ratio	0.9–3.6		–	–	–	–	2.03	–	–	–	–	–	1.41
	Red blood cell (10^12^/L)	4.3–5.8	5.05	4.94	–	4.8	4.53	4.86	–	4.86	–	–	4.53	4.14
	Hemoglobin (g/L)	130–175	158	155	–	151	141	151	–	157	–	–	142	129
	Platelet (10^9^/L)	125–350	207	257	–	324	283	329	–	286	–	–	179	127
Biochemical tests	Albumin (ALB) (g/L)	35–50	39.7	–	39.5	–	–	40.3	–	38.9	37.9	–	41.7	40.7
	Total Bilirubin (TB) (μmol/L)	1.7–21	19	–	17.2	–	–	13.7	–	19.1	14.1	–	8.2	**21.9**
	Direct Bilirubin (DB) (μmol/L)	0.3–6.8	0	–	**7.8**	–	–	6.2	–	**6.9**	5.3	–	3.2	**10.6**
	Alanine aminotransferase (ALT) (U/L)	21–72	65.2	–	51	–	–	**173**	–	**265**	**242**	–	**134**	46
	Aspartate aminotransferase (AST) (U/L)	17–59	59	–	30	–	–	**62**	–	**64**	53	–	31	27
	Alkaline phosphatase (ALP) (U/L)	45–125	**231**	–	**172**	–	–	123	–	95	97	–	96	77
	γ-glutamyl transpeptadase (GGT) (U/L)	0–49	**508**	–	**473**	–	–	**371**	–	**277**	**230**	–	**135**	**104**
	Lactate dehydrogenase (LDH) (U/L)	313–618	**770**	–		–	–	257	–	206	201	–	195	151
	Blood urea nitrogen (BUN) (mmol/L)	3.2–7.1	4.34	–	5.38	–	–	5.17	–	4.29	–	–	1.2	1.66
	creatinine (Cr) (μmol/L)	57–97	61.8	–	55	–	–	61	–	66	–	–	66	57
	cystatin c (Cys-C) (mg/L)	0–1.21	–	–	0.7	–	–	1.17	–	0.91	–	–	0.65	0.5
	B2 microglobulin (Β2-M) (mg/L)	1.0–3.0	–	–	1.46	–	–	0.73	–	1.52	–	–	2.27	2.02
	Procalcitonin (PCT) (ng/mL)	< 0.1	0.053	0.05	0.047	0.039	0.021	0.044	–	0.068	–	–	**0.103**	0.034
	C-reactive protein (CRP) (mg/L)	< 10	**177**	**48.73**	**19.37**	9.23	4.94	3.11	–	7.43	–	–	**11.24**	3.93
	Creatine kinase (CK) (U/L)	55–170	–	–	67	–	–	–	–	–	–	–	–	
	Creatine kinase-MB (CK-MB) (ng/mL)	0–2.37	< 0.22	–	0.76	–	–	–	–	–	–	–	–	0.84
	Myoglobin (MYO) (ng/mL)	0–110	78.3	–	103.63	–	–	–	–	–	–	–	–	25.36
	Cardiac Troponin I (CTnI) (ng/mL)	< 0.04	< 0.012	–	< 0.006	–	–	–	–	–	–	–	–	< 0.006
	type B natriuretic peptide (BNP) (pmol/L)	0–23.1	**34.1**	–		–	–	–	–	–	–	–	–	
	Interleukin-6 (IL6) (pg/mL)	0–7	**16.19**	–		–	–	–	–	–	–	–	–	< 1.5
Blood gas assay	PH	7.35–7.45	7.428	7.448	**7.452**	7.447	7.445	**7.457**	7.432	**7.454**	7.429	7.414	7.395	7.392
	PCO2 (mmHg)	35–45	43	39.1	39.1	39.2	39.7	39	41.8	38.8	41.5	43.3	**45.6**	**45.6**
	PO2 (mmHg)	75–110	64.4	**137**	**137**	101	**113**	74.2	71.3	87.1	75.7	83.8	92.5	104
	Actual sodium bicarbonate (mmol/L)	21.4–27.3	**28.4**	27	27.2	27	27.3	**27.5**	**27.9**	27.2	**27.5**	**27.7**	**27.9**	**27.7**
	Whole blood alkali surplus (mmol/L)	−3-3	**3.4**	2.8	3.2	2.8	3	**3.5**	**3.6**	**3.3**	**3.1**	2.6	3	2.5
	Oxygenation index	400–500	157	304	304	259	323	195	188	194	261	399	319	359
	Lactate (mmol/L)	0.5–1.6	1.4	1.5	1.3	**1.8**	1.3	1.6	1	1.2	1.3	1.5	**2.4**	**2**
	Fractional concentration of inspired oxygen (FiO2) (%)	15.0–100	41	45	45	39	35	38	38	45	29	21	29	29
Blood coagulation function tests	Prothrombin time activity (PTA) (%)	70–120	107	–	98	–	–	–	–	–	**136**	–	–	–
	Prothrombin time (PT) (Sec)	11–15.1	12.7	–	13.2	–	–	–	–	–	11.5	–	–	–
	Prothrombin time International normalizaed ration (PT INR)	0.75–1.25	0.96	–	1.01	–	–	–	–	–	0.85	–	–	–
	Activated partial thromboplastin time (APTT) (Sec)	28–43.5	31.8	–	30	–	–	–	–	–	28.3	–	–	–
	Antithrombin III (AT III) (%)	85–135	100	–	105	–	–	–	–	–	117	–	–	–
	Fibrinogen (FIB) (g/L)	2–4	**7.79**	–	**5.37**	–	–	–	–	–	**4.62**	–	–	–
	D-Dimer (Diffused intravascular coagulation, D-DIC) (μg/mL)	0–0.5	**0.95**	–	0.49	–	–	–	–	–	0.29	–	–	–

**Fig. 1:**
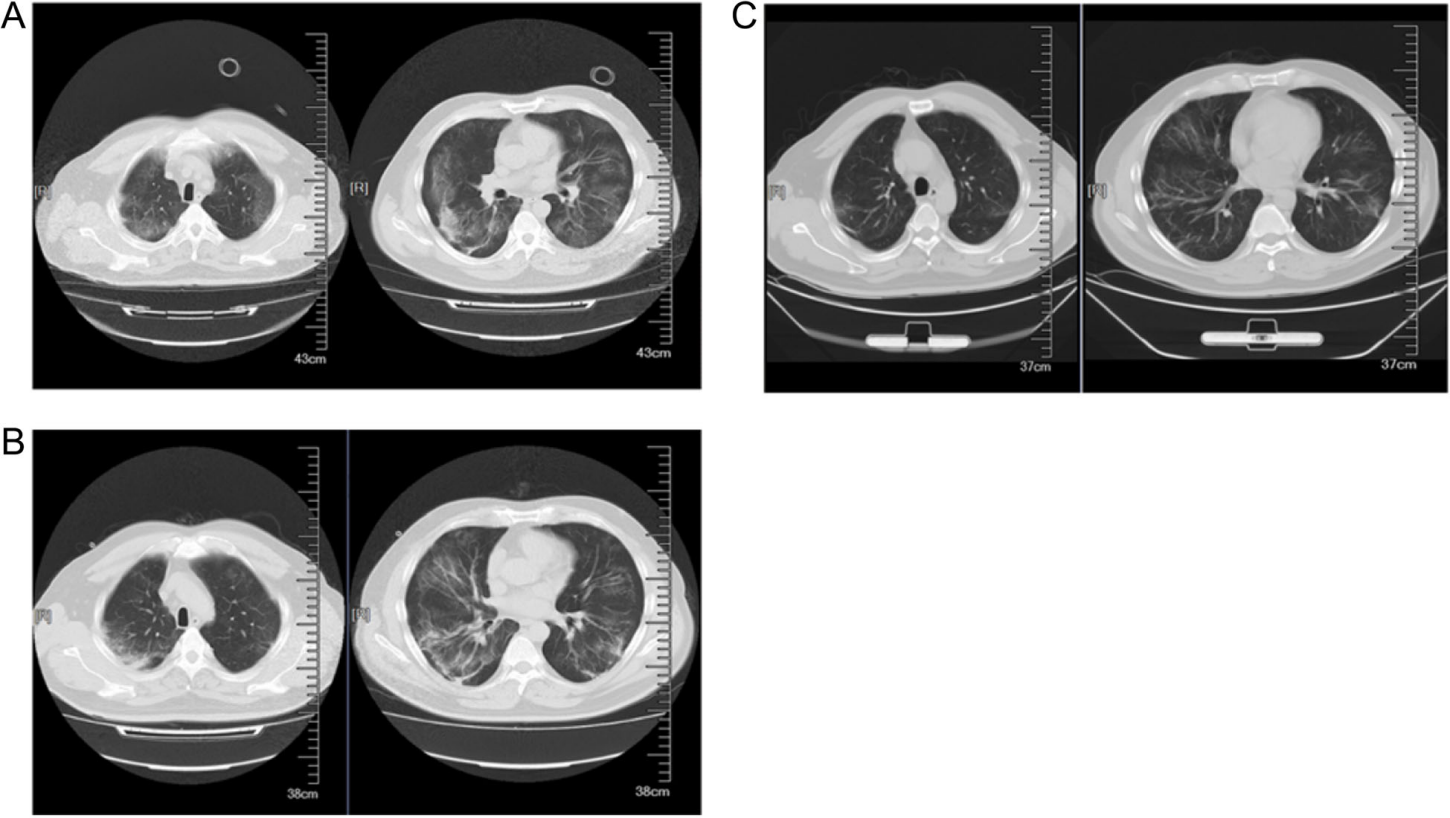
CT scanning results of the lungs. **a**. CT scan images on 25 January. **b**. CT scan images on 29 January. **c**. CT scan images on 12 February. Improvements in abnormalities could be seen

**Fig. 2:**
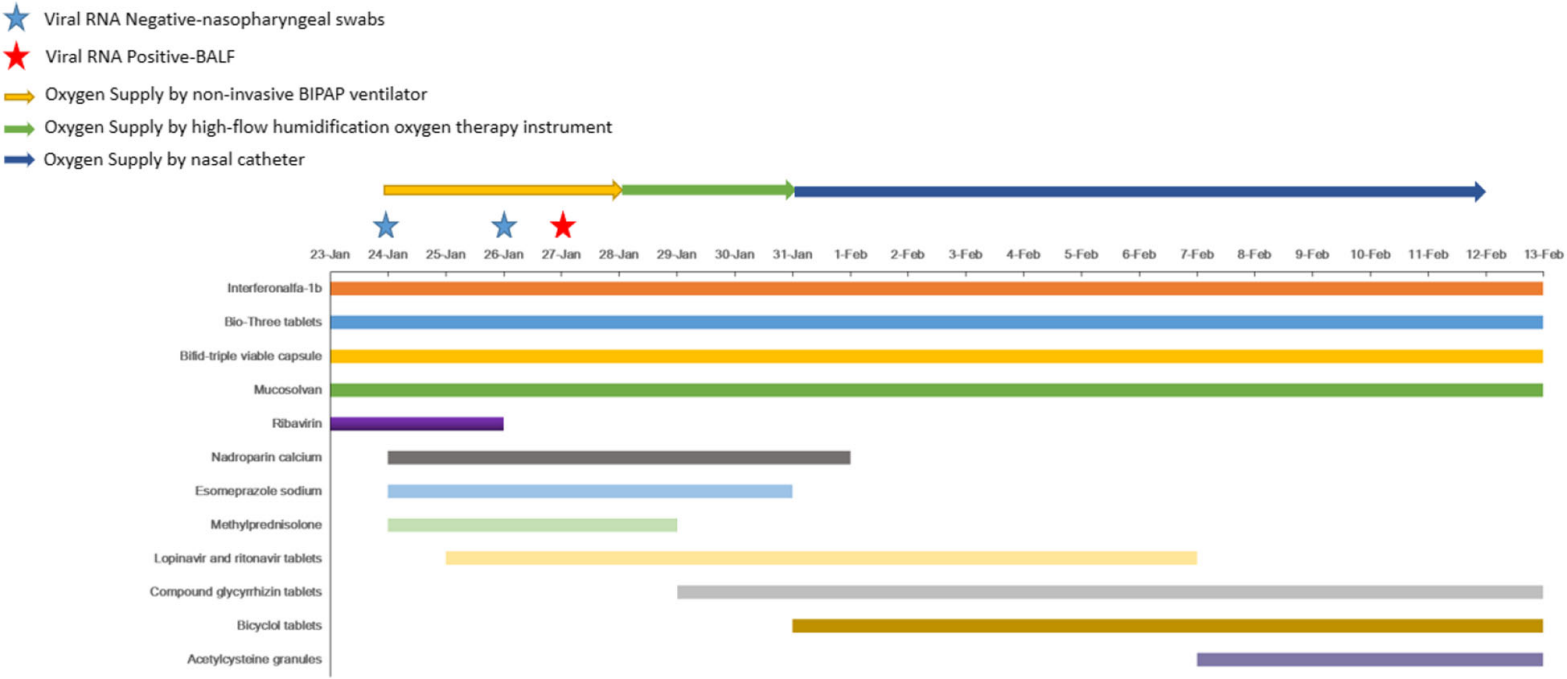
Treatment schemes and Viral RNA detection time points

From 25 January onward, the patient’s syndromes had gradually resolved with only occasional dry cough. Computed tomography (CT) scan of the lungs was performed on 25th, 29th January and 12th February (Fig. 1). Evidence of severe pneumonia, including multiple lesions and swollen lymph nodes, could be seen from both of the lungs on 25th January. (Fig. 1a) The patient was then given 500 mg lopinavir and ritonavir tablets (Abbott S.P.A., Italy) every 12 h until 6 Feb as a combination treatment for antiviral effect. SARS-COV-2 ribonucleic acid test was negative using nasopharyngeal swabs done by our hospital on 26 January. To confirm the presence of SARS-COV-2, the patient’s BALF sample was sent to Shenzhen CDC for viral nucleic acid detection. SARS-COV-2 ribonucleic acid was tested positive for BALF sample on 27 January. Oxygen supplement by non-invasive BIPAP was replaced by high-flow humidification oxygen therapy instrument for higher oxygen flow of 45 L/min and oxygen concentration of 40% on 28 January. Biochemical test results indicated an increased level of alanine aminotransferase (ALT) since 29 January. The patient was diagnosed to have toxipathic hepatitis which was possibly induced by SARS-COV-2. The patient then received 50 mg compound glycyrrhizin tablets (Akiyama Jozai Co., Ltd., Japan) three times per day from 29 January to 13 February and 50 mg bicyclol tablets (Beijing Union Pharmaceutical Factory, China) three times per day from 31 January to 13 February for liver protection. (Figure 2) ALT level resumed to the normal range on 13 February. To prevent pulmonary fibrosis, the patient was given 7-day course of 0.2 g acetylcysteine granules (Bio Pharmacceutical, China) three times per day from 7 February. Obvious improvements could be seen from the subsequent CT scanning results of the lungs on 29th January (Fig. 1b) and 12 February (Fig. 1c). Oxygen supplement by high-flow humidification oxygen therapy instrument was discontinued on 31 January and the patient was given nasal catheter for oxygen inhalation with a flow rate of 4 L/min. His clinical conditions was stable with fractional concentration of inspired oxygen (FiO_2_) value fluctuating within the normal range. (Table 1) On 2 February, the patient stated that there was obvious improvement of his symptoms. CT scanning of the lungs (Fig. 1c) on 12 February confirmed the improvement and the patient was discharged on the following day.

## Discussion and conclusions

This case report described the symptoms of a SARS-COV-2 infected patient and treatment measures taken to the patient. The patient was in close contact with a person from Wuhan and was infected through human-to-human transmission. According to the seventh edition of Novel Coronavirus Pneumonia (NCP) treatment plan issued by National Health Commission of the People’s Republic of China, this patient in the case report is considered as a severely ill case of the COVID-19 pneumonia. The patient’s course of disease reached 11 days which is the period of acme at the time of hospital admission and showed respiratory failure symptoms. The unconventional aspect of this case is that the detection of the patient’s upper respiratory tract specimen was SARS-COV-2 negative repeatedly while that of BALF sample was positive for SARS-COV-2 virus. This suggests that the lung is possibly the main target of the SARS-COV-2 virus. We speculate that the virus migrates from upper respiratory tract to lower respiratory tract as the infection progresses, which may explain the negative test results of nasopharyngeal swabs. As reported by Dr. Yang Yang and his group, the nucleic acid of SARS-COV-2 could be detected from BALF samples of critically ill patients with COVID-19 pneumonia while not from the upper respiratory tract specimens of some patients [10]. In contrast, the detection of SARS-COV-2 RNA was positive from the upper respiratory tract specimens at mild stage of illness [11]. This indicates that the distribution of this virus in the respiratory system is closely related to the severity of the disease, and also indicates that early diagnosis is a key point to avoid false negative results when testing by direct methods. Thus, detection of SARS-COV-2 RNA in BALF samples is of great importance in confirming of the infection in the similar cases to the one described in this report. Although it is largely supportive, our treatment scheme was proven to be effective in helping the patient combating the virus. Our report highlights the importance to carry out bronchoscopy and detection of SARS-COV-2 RNA from BALF samples as complementary interventions in addition to monitoring epidemiological changes, clinical symptoms and chest CT findings of the unconventional COVID-19 pneumonia cases like the one described in this report, which will be informative and clinically significant in guiding the prognosis of the disease.

## Data Availability

All data generated or analysed during this study are included in this published article.

## Abbreviations

COVID-19: Coronavirus disease 2019
SARS-COV-2: Severe acute respiratory syndrome coronavirus 2
BALF: Bronchoalveolar-lavage fluid
CT: Computed tomography
BIPAP: Bilevel positive airway pressure
RSV: Respiratory syncytial virus
RNA: Ribonucleic acid
IPAP: Pressure for inhalation
EPAP: Pressure for exhalation
ESR: Erythrocyte sedimentation rate
DIC: Diffuse intravascular coagulation
CDC: Center for Disease Control
ALT: Alanine aminotransferase
FiO_2_: Fractional concentration of inspired oxygen
